# Semantic Stroop interference is modulated by the availability of executive resources: Insights from delta-plot analyses and cognitive load manipulation

**DOI:** 10.3758/s13421-024-01552-5

**Published:** 2024-03-26

**Authors:** Simone Sulpizio, Giacomo Spinelli, Michele Scaltritti

**Affiliations:** 1 Dipartimento di Psicologia, Università di Milano–Bicocca, Milano, Italy; 2 Milan Center for Neuroscience (NeuroMI), University of Milano–Bicocca, Milano, Italy; 3 Dipartimento di Psicologia e Scienze Cognitive, Università degli Studi di Trento, Trento, Italy

**Keywords:** Visual word recognition, Stroop, Semantic processing, Cognitive control, Working-memory load

## Abstract

We investigated whether, during visual word recognition, semantic processing is modulated by attentional control mechanisms directed at matching semantic information with task-relevant goals. In previous research, we analyzed the semantic Stroop interference as a function of response latency (delta-plot analyses) and found that this phenomenon mainly occurs in the slowest responses. Here, we investigated whether this pattern is due to reduced ability to proactively maintain the task goal in these slowest trials. In two pairs of experiments, participants completed two semantic Stroop tasks: a classic semantic Stroop task (Experiment 1A and 2A) and a semantic Stroop task combined with an *n*-back task (Experiment 1B and 2B). The two pairs of experiments only differed in the trial pace, which was slightly faster in Experiments 2A and 2B than in Experiments 1A and 1B. By taxing the executive control system, the *n*-back task was expected to hinder proactive control. Delta-plot analyses of the semantic Stroop task replicated the enhanced effect in the slowest responses, but only under sufficient time pressure. Combining the semantic Stroop task with the *n*-back task produced a change in the distributional profile of semantic Stroop interference, which we ascribe to a general difficulty in the use of proactive control. Our findings suggest that semantic Stroop interference is, to some extent, dependent on the available executive resources, while also being sensitive to subtle variations in task conditions.

**Supplementary Information:**

The online version contains supplementary material available at 10.3758/s13421-024-01552-5.

The main goal of reading and visual word recognition is to extract meaning from print. Yet a large part of the literature on visual word recognition has considered the retrieval of semantic information as an almost automatic consequence of word recognition (for discussions and alternatives, see Balota & Yap, 2006). This view has been challenged by recent proposals suggesting that semantic representations are not merely accessed from long-term memory stores, but also dynamically processed via cognitive control mechanisms that match semantic information with task- and context-relevant goals (Lambon Ralph et al., 2017; for recent empirical evidence, see, e.g., Montefinese et al., 2020; Scaltritti et al. 2021; Sulpizio et al., 2022).

Cognitive control can be conceptualized as a set of different yet related processes (e.g., Friedman & Miyake, 2004), among which selective attention plays an important role (Kane & Engle, 2003). Using selective attention, we can focus on goal-relevant information, while ignoring distracting (i.e., irrelevant) elements. Considering semantic access, for example, selective attention may be pivotal when dealing with task-relevant but nondominant semantic features of the stimulus (e.g., referring to a whale as a mammal), or with the nondominant meaning of a word (e.g., *bark*, as the covering of a tree). These are relatively frequent events which would critically hinge on cognitive control (e.g., Davey et al., 2016; Hoffman et al., 2018; Lambon Ralph et al., 2017).

A classic way to study cognitive control and selective attention in a laboratory setting is the Stroop paradigm (Stroop, 1935). In the typical task configuration, participants are presented with colored written words, and are asked to name the color while ignoring the word. The distractor word is a color word (e.g., *blue*) and the classic result—the Stroop effect—is that participants are slower (and less accurate) when the word is incongruent with the color response (e.g., the word *blue* written in red) than when the two are congruent (e.g., the word *blue* written in blue). Of particular interest for the present study is the semantic variant of the Stroop task (Klein, 1964), in which distractor words are either strongly associated with a specific color (e.g., *fire*, associated with red) or not (e.g., *book*). When presented in an incongruent ink color (e.g., *fire* in green), color-associated words are slower than color-unrelated words (e.g., *book* in green). This semantic Stroop effect has been considered as a marker of semantic conflict resulting from the activation of different semantic codes (e.g., Augustinova et al., 2018; Neely & Kahan, 2001; but for a different interpretation, see, e.g., Roelofs, 2003). Of importance, this activation would occur despite participants being explicitly instructed to ignore the word because any readable written stimulus is assumed to trigger word reading automatically (MacLeod & MacDonald, 2000; Megherbi et al., 2018).

Although the Stroop effect is regarded as a robust phenomenon (e.g., Hedge et al., 2018), several processes have been found to shape its size and impact, such as the proportion of neutral (e.g., Goldfarb & Henik, 2007; Spinelli & Lupker, 2021) or incongruent trials composing the experimental list (e.g., Hutchison, 2011; Spinelli & Lupker, 2023; Spinelli et al., 2019). These results have been interpreted as evidence of conflict-induced attentional adjustments (e.g., Bugg & Crump, 2012). Attentional adjustments in Stroop tasks have been linked to the proposal that cognitive control is regulated using both (top-down) proactive and (bottom-up) reactive control processes (Braver, 2012). Proactive control processes allow individuals to actively maintain attention focused on task-relevant information in a sustained but effortful fashion. Reactive control processes, instead, allow individuals to relax proactive maintenance, with attention being refocused in a transient fashion only when required by the task (e.g., when task-relevant and task-irrelevant components of the stimulus are in conflict; i.e., with an incongruent Stroop stimulus). In line with these ideas, experimental manipulations aimed at inducing proactive and reactive control have been linked to changes in the size of the Stroop effect: A smaller Stroop effect is observed when proactive, as opposed to reactive, control is induced (Gonthier et al., 2016; Spinelli & Lupker, 2023; see also Spinelli & Lupker, 2021). A potential reason for these changes is that proactive control leads to a reduction in activation for the (irrelevant) word reading task, thus reducing the conflict with the (relevant) color naming task. Differently, when reactive control is the dominant control process, the reading task will remain strongly active and enhance the conflict with the color naming task (e.g., Goldfarb & Henik, 2007; Kalanthroff et al., 2015, 2018).

Apart from manipulations aimed at inducing reactive vs. proactive control, even fluctuations of attention over time would cause a trial-by-trial swinging between proactive and reactive control (e.g., Cohen & Maunsell, 2011; Landau & Fries 2012; Van Rullen et al., 2007), partly because sustaining proactive control is assumed to be effortful. As a result, it would be difficult for participants to attend to the task goal optimally (i.e., naming the color while ignoring the word to the extent possible) in a consistent fashion. Interestingly, situations suggesting suboptimal attention deployment during the semantic Stroop task have been associated with an increase of the semantic Stroop effect. In a recent work, Scaltritti et al. (2022) reported that the semantic Stroop effect was negligible or absent in faster responses while tended to be maximal in the slower ones (for the same pattern, see also Hasshim et al., 2019; Sulpizio et al., 2022). The authors interpreted this pattern as reflecting fluctuations in the ability of attentional control to maintain the task goal in an optimal fashion (i.e., by reducing the impact of distracting information; De Jong et al., 1999; see also San José et al., 2021; Scaltritti et al., 2015). When attention to the task goal is deployed less optimally (a situation that is more likely to have occurred in trials receiving the slowest responses; De Jong et al., 1999), interference phenomena, including semantic ones, would be enhanced because of the greater activation of the task-irrelevant stimulus (i.e., the distractor word). Thus, the larger semantic Stroop effect for slower responses reported by Scaltritti et al. (2022) may reflect the byproduct of reduced proactive control in those trials.

The present study directly tackles this hypothesis by investigating how semantic Stroop interference varies as a function of the availability of executive resources for engaging proactive control. To reach this aim, participants performed two experimental procedures: (a) a single-task procedure, in which participants performed the classic semantic Stroop paradigm (Klein, 1964; Scaltritti et al., 2022); (b) a dual-task procedure, in which the same semantic Stroop paradigm was combined with an *n*-back paradigm, in which a stream of stimuli is presented (in alternation with Stroop trials) and participants have to decide whether the currently presented stimulus is identical to a previous one.

Using the single-task procedure, we have previously found (Scaltritti et al., 2022; Sulpizio et al., 2022) an increase of the semantic Stroop interference as a function of response latency. This pattern is the focus of the present investigation. Also, as in our previous studies, we opted for the manual version of the task, in which responses are delivered by button press (Scaltritti et al., 2022; Sulpizio et al., 2022) and the stimulus (color)-response (button press) mapping is completely arbitrary. Concerning the dual-task procedure, we combined the manual semantic Stroop procedure with an *n*-back task because the latter is well-known to involve the maintenance and updating component of working memory and thus to tax the executive control system (e.g., Chatham et al., 2011; Owen et al., 2005). Further, the *n-*back task has already been used to investigate cognitive control in the context of the (classic) Stroop task (Soutschek et al., 2013), including its manual version (Kalanthroff et al., 2015). We reasoned that, by directly taxing the executive control system, the *n-*back task would reduce the amount of available executive resources, thus reducing the chance to engage proactive control during the Stroop task. The resulting bias towards reactive control mechanisms would thus trigger enhanced interference from conflicting task-irrelevant information.

As mentioned, our focus is on delta-plot analyses, where the effect under examination (i.e., the difference between response latencies for trials featuring color associated vs. color-unrelated carrier words) is assessed across the different quantiles of the RT distribution (e.g., De Jong et al., 1994), thus capturing how the semantic Stroop effect varies as a function of response latency. In the single-task procedure, we expect to replicate our previous results and find a reliable effect in the slower responses, but a negligible (or no) effect in the faster ones (Scaltritti et al., 2022; Sulpizio et al., 2022). This pattern would be ascribed to failures of attentional control in optimally maintaining the task goal for trials with slower responses (e.g., De Jong et al., 1999; San José et al., 2021; Scaltritti et al., 2015), possibly because, in these slower trials, control may be more frequently reactive rather than proactive. In the dual-task procedure, because of the working memory load induced by the *n*-back task (Braver et al., 1997; Owen et al., 2005), there should be fewer resources available to apply proactive control consistently across the whole experiment, with a subsequent increased reliance on reactive control. Therefore, the semantic Stroop effect may also surface in faster responses (although those responses would likely be slower than the fastest responses in the single-task procedure due to concurrent-task costs; e.g., de Fockert et al., 2001). The reason is that the general depletion of executive resources across all trials would hinder the ability to focus on the task goal consistently across trials (rather than determining episodic lapses and failures of attention mostly reflected in the slowest latencies), thus allowing semantic interference to have an impact across the whole RTs distribution. However, this impact may be possibly further enhanced in the slower responses of the dual-task procedure, in which lapses and failures would occur with a higher frequency than on the other trials, reflecting a pronounced difficulty to engage proactive control.

Although our main interest focuses on delta-plot analyses, we also explored the overall semantic Stroop effect. Because our prediction, following Scaltritti et al. (2022), was that a concurrent working memory load would reduce the number of episodes in which proactive control is fully operative, it follows that a larger semantic Stroop effect should be expected. This prediction appears to be in line with findings from Stroop-like tasks showing that congruency effects are smaller in single (i.e., under no load) than in dual task conditions, and smaller under low than high load in dual tasks (e.g., de Fockert et al., 2001; Lavie et al., 2004). Those findings suggest that interference from task-irrelevant information (potentially including semantic information, although those studies did not focus on this specific type of interference) is better handled in conditions featuring no cognitive load, or a reduced one, thus allowing to engage proactive control with ease. However, when cognitive load is introduced, proactive control will be more difficult to engage, and interference phenomena (potentially including semantic ones) will have a larger overall impact.

Other studies, instead, suggest a different scenario. Kalanthroff et al. (2015) ran a study using a dual-task procedure involving the classic manual Stroop task in combination with a letter-variant of the *n-*back task. In the latter task, participants were presented with letters (in alternation with Stroop trials) and, for each one of them, had to decide whether the current letter was identical to a previously presented one. In one condition they used a low working memory load (0-back condition; i.e., participants simply had to press a button when a specific letter was presented), whereas in the other condition they used a high load (2-back, i.e., participants had to press a button if the current letter was identical to the one presented two trials before). Concerning the Stroop implementation, across both working memory load conditions, they used a congruent (*red* written in red ink) and an incongruent condition (*blue* written in red ink)—which are both associated with task conflict because they involve a readable written stimulus (i.e., a color name)—as well as a neutral condition (i.e., a letter string in Hebrew comparable to a series of X in English)—a condition which is not associated with task conflict because it does not involve a readable written stimulus. Interestingly, they found that, compared with the low-load condition, the high load increased the response latencies for congruent and incongruent stimuli to a similar extent (i.e., the size of the Stroop effect remained unchanged), with a reduced influence on neutral stimuli. Congruent stimuli in the high load condition thus became *slower* than neutral stimuli, a reversal of the typical facilitation pattern (i.e., congruent faster than neutral). According to Kalanthroff et al., these findings suggest that cognitive load mainly impacts the ability to engage proactive control, hindering the ability to deal with the *task* conflict produced by congruent and incongruent (but not neutral) stimuli.

A corollary of this explanation is that the Stroop effect, being a contrast between stimuli which both involve task conflict, would be essentially unaffected by a dual task procedure, as indeed observed in Kalanthroff et al. with respect to the classic Stroop effect. Under this perspective, a similar prediction can be considered for the semantic Stroop variant as well (i.e., a null impact of a dual task procedure), as the semantic Stroop effect is also based on a contrast between two readable written stimuli (e.g., *fire* in green and *book* in green). Note, however, that Kalanthroff et al. did not investigate semantic interference, a type of interference that is only one of the components of the Stroop effect (Parris et al., 2022). Therefore, whether the semantic Stroop effect is affected or not by a concurrent working memory load is an open issue that our results will contribute to clarify empirically.

## Experiment 1A (single-task procedure) and 1B (dual-task procedure)

### Method

#### Participants

Sample size was estimated based on our previous studies investigating semantic Stroop interferences (Scaltritti et al., 2022; Sulpizio et al., 2022). This sample was also more than 4 times larger than previous studies combining the Stroop task with the *n*-back task (Kalanthroff et al., 2015; Soutschek et al., 2013).

Ninety-nine Italian native speakers took part in both Experiment 1A and Experiment 1B (40 females, mean age = 27.26 years, *SD* = 4.82, range: 20–40 years). Six participants were recruited among direct contacts of the authors, whereas 93 participants were recruited via the research platform Prolific Academic (Palan & Schitter, 2018) and rewarded with £5.60. All participants reported normal or corrected-to-normal vision and no history of learning disabilities. Four participants were excluded due to many missing trials in the data (>20%) and four because of the low accuracy in their overall performance (>3 *SD* below the sample mean). The final sample thus consisted of 91 participants. The study was approved by the Ethical Committee of the University of Milano-Bicocca (protocol n.: RM-2020-279).

#### Stimuli

For the semantic Stroop task, four concrete color-associated words were selected, *prato* (lawn), *fragola* (strawberry), *cielo* (sky), and *limone* (lemon). These items were the same used in our previous semantic Stroop experiments (Scaltritti et al., 2022; Sulpizio et al., 2022). Four concrete words not associated with colors were selected to serve as control stimuli. These were *mazzo* (deck), *cratere* (crater), *bagno* (bath), and *salita* (hill) (the same as in Scaltritti et al., 2022). Control words were matched, as closely as possible, with color-associated words on several psycholinguistic variables (see Table 1). The words we used did not share their initial phonemes with the names of the colors involved in the color-associated words—*verde* (green), *rosso* (red), *azzurro* (light blue), and *giallo* (yellow). Color-associated words were presented only in combination with incongruent colors (e.g., *prato* [lawn] was presented only in red, light blue, and yellow). Likewise, each corresponding control word appeared only in three colors (e.g., *mazzo* [deck], the control word for *prato* [lawn], was presented only in red, light blue, and yellow).

**Table 1:** Psycholinguistic properties of the words in the semantic Stroop task

Variables	Color-associated	Control
Freq. (log)	7.21	6.99
*N* of letters	5.75	5.75
*N* of syllables	2.50	2.50
Orth. *N*	5.25	6.50
OLD	1.71	1.63

*Note*. Freq. (log) = log-transformed lexical frequency; *N* of letters = number of letters; *N* of syllables = number of syllables; Orth. *N* = number of orthographic neighbors; OLD = orthographic Levenshtein distance (Yarkoni et al., 2008). Frequency values (log-transformed) were taken from the SUBTLEX-IT database (Crepaldi et al., 2013). Number of orthographic neighbors and OLD were computed on the PhonItalia database (Goslin et al., 2014) using the vwr package (Keuleers, 2013) in R. Statistical comparisons were not conducted due to the low number of items (4) in each category

For the *n*-back task, we used the same five letters (B, D, G, P, and T) used by previous works combining the Stroop and the *n*-back task (Kalanthroff et al., 2015; Soutschek et al., 2013).

#### Apparatus and procedure

The experiments were programmed with the Open Sesame software (Version 3.2.8; Mathôt et al., 2012) and were administered online, using JATOS (Version 3.5.8; Lange et al., 2015) to manage data collection. At the beginning of the experiment, participants were asked to close all the other windows in the browser and all the other applications, as well as to set the browser to full screen mode. They were then presented with an informed consent screen and asked whether they wanted to proceed. After acceptance, participants provided demographic information (age and gender) and were directed to the first experimental procedure.

Each participant performed both the single (semantic Stroop) task (Experiment 1A) and the dual (semantic Stroop and *n*-back) task (Experiment 1B) procedure. The order of administration was counterbalanced across participants.

**Fig. 1:**
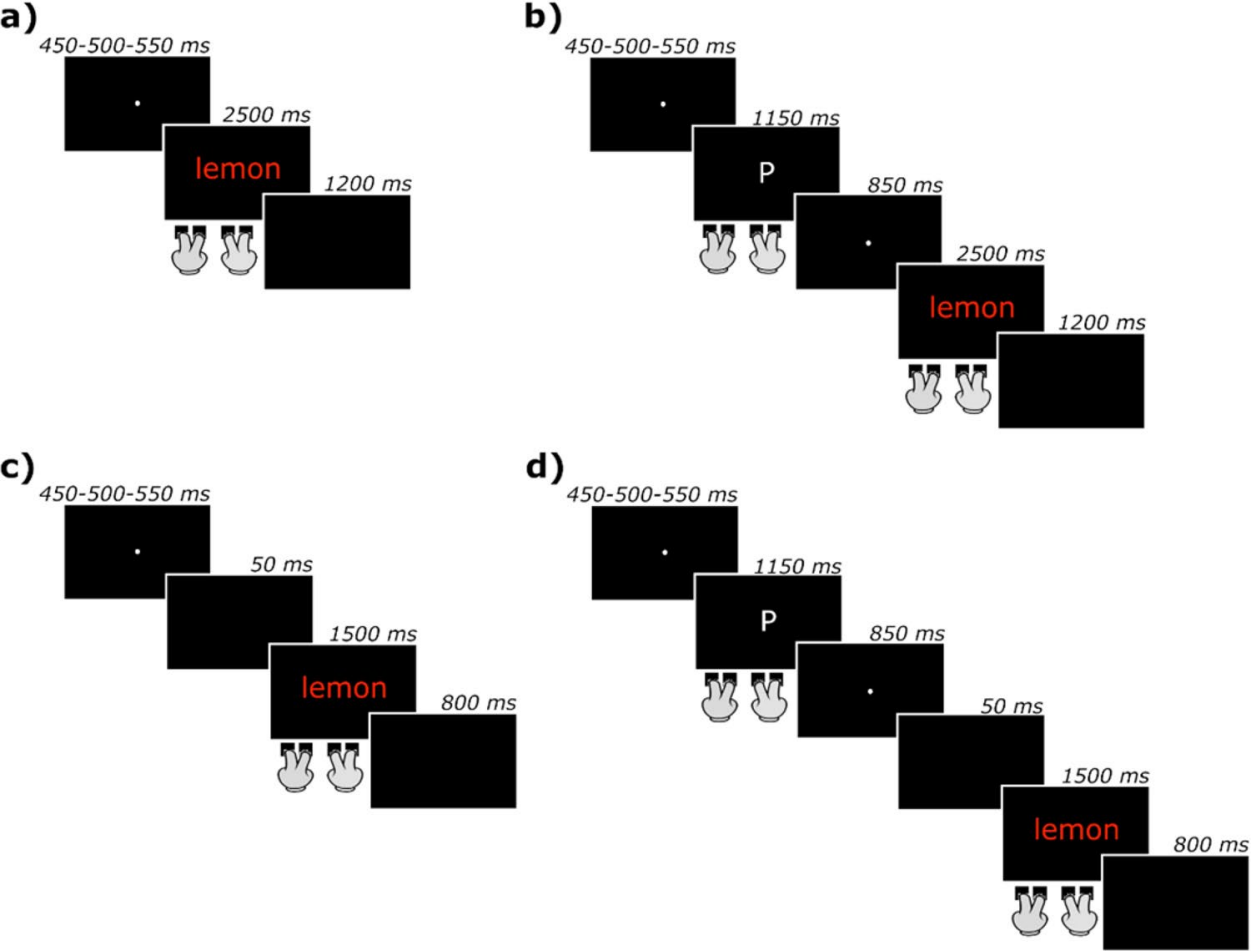
Schematic representations of the procedures used in Experiments 1A and 1B (**a, b**) and 2A and 2B (**c, d**). The words used in the experiment were in Italian, not in English. When participants failed to respond within the allotted time for the Stroop task, a feedback screen reading “*troppo lento!*” (“too slow!”) was displayed for 300 ms immediately following the stimulus screen. (Color figure online)

In the single-task (see Fig. 1a), participants were instructed to categorize the color in which word stimuli were written by pressing one of four buttons (red = Z; yellow = X; green = N; light blue = M), using their right and left index and middle fingers (one finger per response button). Each trial started with a fixation cross presented at the center of the screen for 450, 500, or 550 ms (randomly sampled). Next, the target stimulus (a colored word) was displayed until response. When participants failed to respond within the allotted time (2,500 ms), a feedback screen reading “*troppo lento!*” (“too slow!”) was displayed for 300 ms. The beginning of the next trial occurred after a blank screen lasting 1,200 ms.

The dual-task procedure (Fig. 1b) was modelled after the study by Kalanthroff et al. (2015). Specifically, participants were instructed to (a) categorize the color in which word stimuli were written (semantic Stroop task), and (b) press a button if the currently presented letter was identical to that presented two trials earlier (*n*-back task). For the semantic Stroop task, responses were delivered as in the single task. For the *n*-back task, participants could press any of the four buttons they used for the semantic Stroop task to give a YES response (i.e., they only had to make a response when the presented letter was identical to the one presented two trials earlier, otherwise they were not supposed to make any response). Each trial started with a fixation cross presented at the center of the screen for 450, 500, or 550 ms (randomly sampled). Next, a letter was displayed for 1,150 ms, during which participants could deliver their response. Then, a fixation cross was presented at the center of the screen for 850 ms and was followed by a colored word displayed until response. When participants failed to respond within the allotted time (2,500 ms), a feedback screen (“*troppo lento!*,” “too slow!”) was displayed for 300 ms. The beginning of the next trial occurred after a blank screen lasting 1,200 ms. No feedback was given for wrong responses in the semantic Stroop task or for wrong and missed responses in the *n*-back task.

We decided to compare the dual-task procedure with a single-task procedure, as we deemed the latter to be the most appropriate baseline. A dual task procedure with lower or no working memory demands (e.g., a Stroop task combined with 1-back or a 0-back task, respectively) would have implied the coordination of two different tasks, thus still hinging on executive resources and triggering corresponding processing costs (Lavie et al., 2004). Differently, our aim was to assess variations dependent on cognitive load with respect to the classic semantic Stroop task configuration: Other than being more directly comparable to our previous results (Scaltritti et al., 2022; Sulpizio et al., 2022), in this implementation no additional source of cognitive requirements is present, except for those assumedly recruited by the Stroop task itself, thus offering a more interpretable baseline.

In the semantic Stroop task, for both the single- and the dual-task procedures, each of the words (i.e., the four color-associated and four control words) was presented 8 times in each of the three possible colors, for a total of 192 trials, which were randomly presented in two blocks of 96 trials each. Each color-word combination occurred equally often across the two blocks. Between the two blocks, participants could take a self-terminated break. In the *n*-back, letters were randomly presented using a sampling-with-replacement randomization procedure. In this way, each letter had an equal probability to appear in any given trial and, throughout the experiment, all letters were presented in similar proportions.

Participants performed a general practice session at the beginning of the whole experiment, and two experiment-specific practice sessions, one at the beginning of each experiment. The general practice session consisted of a response mapping training (Kinoshita et al., 2018; Scaltritti et al., 2022; Sulpizio et al., 2022), in which the stimuli consisted of colored strings of 6 hash-marks (######) and participants had to respond to their color. Each color was presented 4 times, for a total of 16 trials. In the two experiment-specific practice sessions, four words were presented, all different from those used in the experiment proper. Participants were asked to respond to the color in which words were written. Each word appeared 2 times in 3 of the 4 colors, for a total of 24 trials. The only difference between the two experiment-specific practice sessions was that at the beginning of the dual-task experiment, participants were also presented with letters appearing before the words and were asked to respond to them. In all practice sessions, the trial procedure was the same as in the respective experiments, except for the fact that a feedback screen (300 ms) was delivered not just when participants failed to respond within the allotted time, but also in case of incorrect responses to colored words (“*ERRORE,*” error). To facilitate learning of the color-response association, during all practice sessions four small colored squares were constantly displayed in the lower part of the screen, in spatial correspondence to the associated response buttons (on the left side, red = Z, yellow = X; on the right side, green = N, light blue = M).

#### Statistical analyses

Reaction times (RTs) were analyzed with linear mixed effects models and response accuracy with generalized linear mixed effects models, using the lme4 library (Version 4_1.1-21; Bates et al., 2015) in R (R Core Team, 2023). Participants and target colors were included as random intercepts. To assess fixed effects, likelihood ratio tests were used to compare models in which the fixed effect under examination was present versus absent. Fixed terms were retained only when their inclusion determined a significant increase in explained variance. In case any interaction resulted significant, all the involved lower-order terms were retained. For significant effects, effect size was calculated with the *effectsize* library^1^ (Version 0.8.6; Ben-Shachar et al., 2020). In all analyses, we first examined semantic Stroop effects separately for the two experiments. Then, we jointly analyzed the two experiments to test the statistical reliability of any difference between them.

For RTs, analyses were conducted on correct responses. Responses faster than 200 ms were excluded from the analyses as they were considered anticipations. For accuracy, analyses were conducted on the whole dataset and paralleled those for the RT analyses. Response accuracy was modeled as a binomial variable.

For RTs, our main focus was on delta-plot analyses capturing variations of the semantic Stroop effects as a function of response latency. To this aim, within each participant and within each condition, RTs were partitioned into five quantiles: The first quantile included the fastest 20% of responses, the second quantile the next fastest 20%, and so on, until the fifth quantile, which included the slowest 20% of the responses. To assess changes in semantic Stroop effects as a function of response latency, the variable quantile was considered as a numerical fixed effect in the statistical models, and we assessed its potential interaction with the Stroop conditions (color-associated vs. neutral).

For the *n*-back task, we also calculated each participant’s overall accuracy in correctly categorizing the letters, and the *d*-prime (using the *dprime* function in the *psycho* package; Makowski, 2018), which reflects participants’ ability to correctly categorize the presented letters. The first two trials of each block were discarded from the analyses.

### Results

#### Overall effects

The mean RTs and response accuracy for each condition are presented in Table 2.

**Table 2:** Mean response latencies (RTs in ms) and proportion of accurate responses in Experiments 1A (single task) and 1B (dual task)

	RTs		Accuracy	
Condition	*M*	*SE*	*M*	*SE*
Single task				
Semantic	704	11.30	.96	.003
Control	699	11.58	.96	.003
Difference	5		0	
Dual task				
Semantic	863	13.01	.93	.005
Control	866	13.53	.93	.005
Difference	−3		0	

*Note*. *M* = mean; *SE* = standard error of the mean

The analysis of RTs did not show any semantic Stroop effect, either in the single-task experiment, χ^2^(1) = 1.71, *p* = .19, or in the dual-task experiment, χ^2^(1) = 0.28, *p* = .59. The joint analysis of the two experiments revealed that only the effect of experiment was significant, χ^2^(1) = 3224.70, *p* < .001, η_p_^2^ = 0.09: Participants were slower in the dual- than in the single-task experiment (*b* = 180.49, *SE* = 3.10, *t* = 58.15). Neither the effect of semantic Stroop, χ^2^(1) = 0.0, *p* = .91, nor the interaction, χ^2^(1) = 2.19, *p* = .13, were significant.

Response accuracy failed to show any reliable semantic Stroop effect either in the single-task experiment, χ^2^(1) = 0.25, *p* = .87, or in the dual-task experiment, χ^2^(1) = 1.46, *p* = .22. In the joint analysis of the two experiments, only the effect of experiment was significant, χ^2^(1) = 136.88, *p* < .001, with participants being less accurate in the dual- than in the single-task experiment (*b* = −0.57, *SE* = .04, *z* = −11.61). Neither the effect of semantic Stroop, χ^2^(1) = 0.72, *p* = .39, nor the interaction, χ^2^(1) = 0.75, *p* = .38, were significant.

The overall proportion of correct responses in the n-back task was .82, revealing good performance considering the task difficulty. Regarding sensitivity (*d′*), participants showed good ability to detect letters (one-sided *t* test against zero: Mean *d′* = 0.65, *SE* = .03), *t*(90) = 18.40, *p* < .001.

#### Delta-plot analyses

**Fig. 2:**
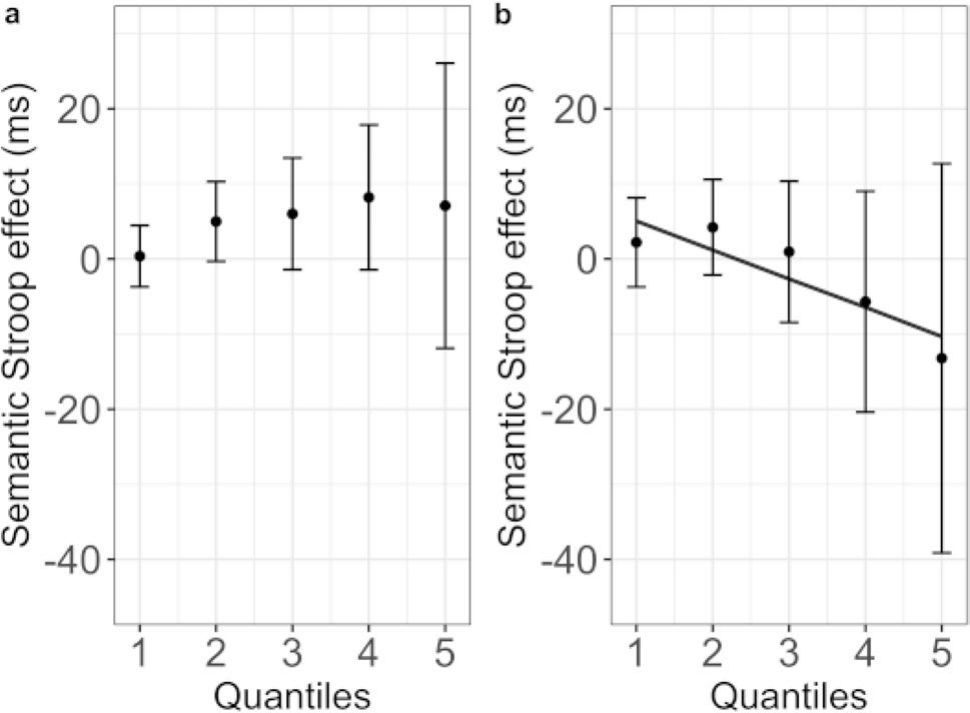
Results of the delta-plot analyses for Experiments 1A (single task) and 1B (dual task). Mean semantic Stroop effect (color-associated—control; *y*-axis) as a function of trial quantile (*x*-axis) for the single-task (**a**) and the dual-task (**b**) procedure. Points represent empirical means, and error bars reflect corresponding 95% confidence intervals. The black line for the dual-task procedure represents the tendency for the reduction of the semantic Stroop effect predicted by the statistical model (Table 3). The absence of any line in the single-task procedure reflects the absence of the interaction between the Stroop effect and quantiles

For the single-task experiment, there were significant effects of semantic Stroop, χ^2^(1) = 4.90, *p* = .02, and quantile, χ^2^(1) = 16076.30, *p* < .001. The interaction was not significant, χ^2^(1) = 1.00, *p* = .31. Parameters of the final model are listed in Table 3 (whereas mean RTs for each condition and quantile are reported in the Supplementary Materials 1, Table S1). As can be seen in Fig. 2a, the semantic Stroop interference is consistently small across quantiles.^2^

**Table 3:** Parameters of the model for the delta-plot analysis for the semantic Stroop task in Experiment 1A (single-task procedure)

Random effects	Variance	*SD*		
Participant	11,516.13	107.31		
Color	40.41	6.35		
Residual	21,461.61	146.49		
Fixed effects	*b*	*SE*	*t*	*η*_*p*_^*2*^
Intercept	299.20	12.04	24.83	
Condition (color-associated)	5.01	2.26	2.21	0.00029
Quantile	133.89	0.81	164.87	0.65

*Note*. *SD* = standard deviation ; *SE* = standard error. Fixed effects were considered as significant when their corresponding *t* value was larger than |2|

For the dual-task experiment, the random intercept for colors had 0 variance, and was thus dropped to aid models’ convergence. The interaction between quantile and experimental condition (control vs color-associated) was significant, χ^2^(1) = 5.14, *p* = .02. Parameters of the final model are listed in Table 4. As can be seen in Fig. 2b, the small semantic Stroop interference in the faster quantiles turns into a facilitation for color associated words in the slower ones, possibly accounting for the absence of an overall effect of the experimental condition in this experiment.

**Table 4:** Parameters of the model for the quantile analysis for the semantic Stroop task in Experiment 1B (dual-task procedure)

Random effects	Variance	*SD*		
Participant	15,300	123.7		
Residual	24,261	155.8		
Fixed effects	*b*	*SE*	*t*	*η*_*p*_^*2*^
Intercept	326.28	13.57	25.50	
Condition (color-associated)	9.40	5.68	1.65	
Quantile	173.82	1.21	143.23	0.56
Condition × Quantile	−3.88	1.71	−2.26	0.00031

*Note*. *SD* = standard deviation; *SE* = standard error. Fixed effects were considered as significant when their corresponding *t* value was larger than |2|

The joint analysis of the two experiments confirms the different distributional profile of the semantic Stroop effect across the procedures, showing a significant three-way interaction between quantile, experimental condition, and experiment, χ^2^(1) = 4.83, *p* = .02. Parameters of the final model are reported in Table 5.

**Table 5:** Parameters of the model for the delta-plot analysis of the semantic Stroop task in both Experiments 1A (single task) and 1B (dual task)

Random effects	Variance	*SD*		
Participant	10,575.35	102.84		
Color	5.90	2.43		
Residual	25,595.95	159.99		
Fixed effects	*b*	*SE*	*t*	*η*_*p*_^*2*^
Intercept	299.63	11.58	25.85	
Condition (color-associated)	0.42	5.75	0.07	
Task (dual)	48.03	5.79	8.28	0.0028
Quantile	133.59	1.23	108.44	0.30
Condition × Task	8.33	8.19	1.01	
Condition × Quantile	1.63	1.73	0.94	
Task × Quantile	39.88	1.75	22.79	0.02
Condition × Task × Quantile	−5.43	2.47	−2.19	0.00014

*Note*. *SD* = standard deviation; *SE* = standard error. Fixed effects were considered as significant when their corresponding *t* value was larger than |2|

### Discussion

The results of the delta-plot analyses revealed some intriguing yet unexpected results suggesting that working memory load modulates semantic Stroop interference differently as a function of response latency. However, before discussing such findings, further investigation is warranted. In fact, the single-task procedure failed to replicate our previous results (Scaltritti et al., 2022; Sulpizio et al., 2022) as it showed neither the semantic Stroop effect in the overall RT analysis, nor its increase as a function of response speed in the delta-plot analysis (an overall semantic Stroop effect did emerge in the delta-plot analysis, but it was quite small). Note, however, that the procedure we used in this pair of experiments, based on Kalanthroff et al.’s (2015), was somewhat different than that in Scaltritti et al. (2022) and Sulpizio et al. (2022). Therefore, we ran a second pair of experiments in which, for the trial structure of the semantic Stroop task, we used exactly the same procedure we adopted in our previous works. The main differences compared with the present Experiments 1A and 1B was the time allotted for responding to Stroop stimuli (1,500 ms instead of 2,500) and the duration of the interstimulus interval (ISI; 800 ms instead of 1,200). In our previous works, the trial pace was a bit faster and, importantly, the time allotted for a response was significantly reduced compared with that used in the present experiments. All the other details were identical to the present Experiments 1A and 1B. With this procedure, we thus expected to detect both the semantic Stroop effect, as well as its prominence in the slowest responses, at least in the single-task procedure.

## Experiment 2A (single-task procedure) and 2B (dual-task procedure)

### Method

#### Participants

Ninety-two Italian native speakers took part in both experiments 2A and 2B (52 females, *M* age = 25.47 years, *SD* = 5.63, range: 22–44 years), all recruited via the research platform Prolific Academic (Palan & Schitter, 2018), and rewarded with £ 6.45. All participants reported normal or corrected-to-normal vision and no history of learning disabilities. One participant was excluded due to an excessive number of missing trials (>20%) and two others because of the low overall accuracy (<3 *SD* from the sample mean). The final sample thus consisted of 89 participants.

#### Stimuli

Stimuli were the same as in Experiment 1.

#### Apparatus and procedure

The apparatus and procedure were almost identical to those of Experiment 1. The only exceptions concerned the duration of some trial events (Fig. 1c and d). Specifically, the maximum allotted time to categorize colored words was 1,500 ms and the ISI lasted 800 ms; also, a short blank (50 ms) was added between the fixation point and the stimulus. The sequence of events and their durations was thus identical to Scaltritti et al. (2022) and Sulpizio et al. (2022).

#### Statistical analyses

The analyses were the same as in Experiment 1.

### Results

#### Overall effects

The mean RTs and response accuracy for each condition are presented in Table 6.

**Table 6:** Mean response latencies (RTs in ms) and proportion of accurate responses in the two experiential procedures

	RTs		Accuracy	
Condition	*M*	*SE*	*M*	*SE*
Single task				
Semantic	662	7.17	.93	.005
Control	653	7.24	.94	.005
Difference	9		−.01	
Dual task				
Semantic	777	9.20	.90	.005
Control	775	9.57	.90	.005
Difference	2		0	

*Note*. *M* = mean; *SE* = standard error of the mean

In the RT analyses of the single-task experiment, a significant effect of semantic Stroop emerged, χ^2^(1) = 9.67, *p* = .001, with participants being slower with color-associated than with neutral words (*b* = 8.86, *SE* = 2.84, *t* = 3.11, η_p_^2^ = 0.00060). In contrast, no semantic Stroop effect emerged in the dual-task experiment, χ^2^(1) = 1.46, *p* = .22. The joint analysis of the two experiments showed that the effect of experiment was significant, χ^2^(1) = 3057.87, *p* < .001, η_p_^2^ = 0.09—with participants being slower in the dual- than in the single-task experiment (*b* = 120.97, *SE* = 2.13, *t* = 56.57)—as was the semantic Stroop effect, χ^2^(1) = 8.44, *p* = .003, η_p_^2^ = 0.00026—with participants being slower in the color-associated than in the neutral condition (*b* = 6.19, *SE* = 2.13, *t* = 2.90). There was, however, no significant interaction between the two factors, χ^2^(1) = 1.61, *p* = .20.

In terms of response accuracy, the semantic Stroop effect was not significant either in the single-task experiment, χ^2^(1) = 0.42, *p* = .51, or in the dual-task experiment, χ^2^(1) = 0.14, *p* = .70. The joint analysis of the two tasks revealed a significant effect of experiment, χ^2^(1) = 128.77, *p* < .001, with participants being less accurate in the dual-task than in the single-task experiment (*b* = −0.47, *SE* = 0.04, *z* = −11.29). No further effect was significant, semantic Stroop: χ^2^(1) = 0.49, *p* = .48; interaction: χ^2^(1) = 0.06, *p* = .79.

The overall proportion of correct responses in the n-back task was .81, revealing good performance. Regarding sensitivity (*d′*), participants showed good ability to detect letters (one-sided* t* test against zero: Mean *d′* = 0.55, *SE* = .02), *t*(88) = 24.91, *p* < .001.

#### Delta-plot analyses

**Fig. 3:**
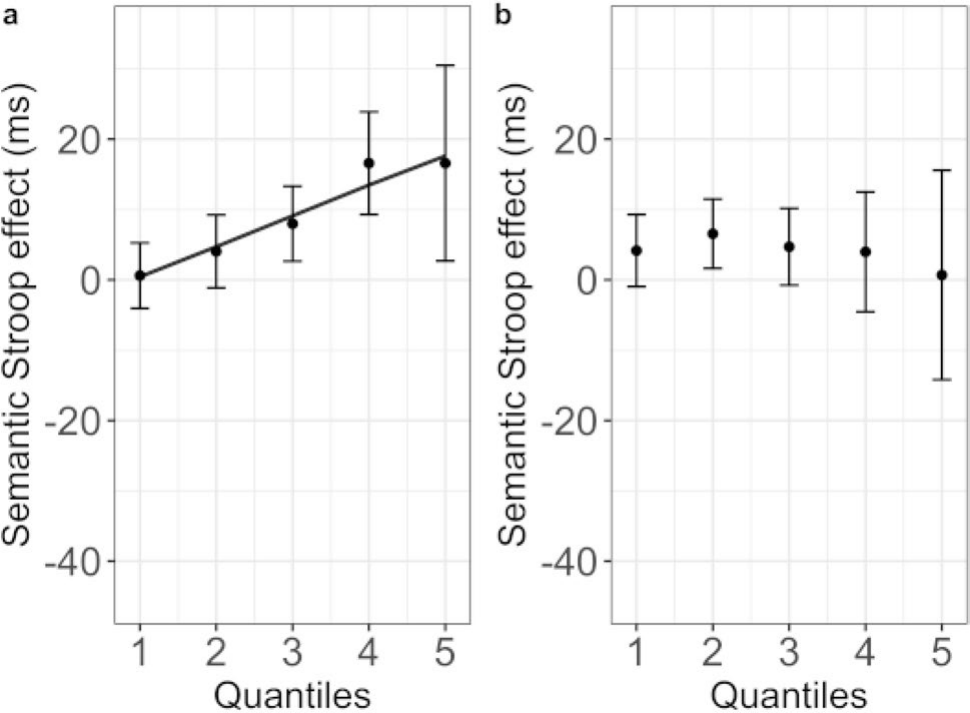
Results of the delta-plot analyses for Experiments 2A (single task) and 2B (dual task). Mean semantic Stroop effect (color-associated—control; *y*-axis) as a function of trial quantile (*x*-axis) for the single-task (**a**) and the dual-task (**b**) procedure. Points represent empirical means, and error bars reflect corresponding 95% confidence intervals. The black line for the single-task procedure represents the tendency for an increase in the semantic Stroop effect predicted by the statistical model. The absence of any line in the dual-task procedure corresponds to the absence of the interaction between the Stroop effect and quantiles

For the single-task experiment, there was a significant interaction between quantile and experimental condition (control vs. color-associated) χ^2^(1) = 18.28, *p* < .001. Parameters of the final model are reported in Table 7 (mean RTs for each condition and quantile are reported in the Supplementary Materials 1, Table S2). As visible in Fig. 3a, the semantic Stroop effect was larger in slower quantiles.

**Table 7:** Parameters of the model for the delta-plot analysis for the semantic Stroop task in Experiment 2A (single-task procedure)

Random effects	Variance	*SD*		
Participant	4,398.87	66.32		
Color	23.56	4.85		
Residual	8,503.73	92.21		
Fixed effects	*b*	*SE*	*t*	*η*_*p*_^*2*^
Intercept	328.68	7.81	42.03	
Condition (color-associated)	−4.06	3.39	−1.19	
Quantile	108.38	0.73	148.45	0.58
Condition × Quantile	4.37	1.02	4.27	0.0011

*Note*. *SD* = standard deviation; *SE* = standard error. Fixed effects were considered as significant when their corresponding *t* value was larger than |2|

For the dual-task experiment, there were significant effects of semantic Stroop, χ^2^(1) = 7.40, *p* = .006, and quantile, χ^2^(1) = 23927.10, *p* < .001; however, the interaction was not significant, χ^2^(1) = 1.47, *p* = .22. Parameters of the final model are listed in Table 8. A small semantic Stroop effect appears across (approximately) all quantiles (Fig. 3b).

**Table 8:** Parameters of the model for the delta-plot analysis for the semantic Stroop task in Experiment 2B (dual-task procedure)

Random effects	Variance	*SD*		
Participant	7,607.25	87.22		
color	7.53	2.74		
Residual	7,863.51	88.67		
Fixed effects	*b*	*SE*	*t*	*η*_*p*_^*2*^
Intercept	402.20	9.52	42.21	
Condition (color-associated)	3.86	1.42	2.71	0.00047
Quantile	124.89	0.51	241.77	0.91

*Note*. *SD* = standard deviation; *SE* = standard error. Fixed effects were considered as significant when their corresponding *t* value was larger than |2|

Critically, in the joint analysis of the two tasks, the three-way interaction between quantile, experimental condition, and task was significant, χ^2^(1) = 13.53, *p* < .001. Parameters of the final model are reported in Table 9.

**Table 9:** Parameters of the model for the delta-plot analysis of the semantic Stroop task in both Experiments 2A (single task) and 2B (dual task)

Random effects	Variance	*SD*		
Participant	4,932.51	70.23		
Color	14.15	3.76		
Residual	9,225.99	96.05		
Fixed effects	*b*	*SE*	*t*	*η*_*p*_^*2*^
Intercept	327.89	8.07	40.59	
Condition (color-associated)	−4.04	3.53	−1.14	
Task (dual)	72.93	3.56	20.48	0.01
Quantile	108.49	0.75	143.26	0.40
Condition × Task	11.24	5.03	2.23	0.00015
Condition × Quantile	4.37	1.06	4.10	0.00053
Task × Quantile	16.78	1.07	15.60	0.0076
Condition × Task × Quantile	−5.59	1.52	−3.67	0.00043

*Note*. *SD* = standard deviation; *SE* = standard error. Fixed effects were considered as significant when their corresponding *t* value was larger than |2|

## General discussion

The present study investigated whether the semantic Stroop interference varies as a function of the availability of resources to engage proactive control. In particular, the availability of fewer executive resources was expected to be associated with a higher difficulty in engaging proactive control in the Stroop task, resulting in enhanced interference across the RT distribution from conflicting task-irrelevant information. To test this, we ran two pairs of experiments in which the same participants performed semantic Stroop tasks in isolation (Experiments 1A and 2A) and in combination with an n-back task taxing the executive control system (Experiments 1B and 2B). Concerning the semantic Stroop task, the only difference between the two pairs of experiments was in the timing of trial events, with a longer ISI and more time allotted for responses in Experiments 1A and 1B—in which Kalanthroff et al.’s (2015) procedure was adopted—than in Experiments 2A and 2B—in which Scaltritti et al.’s (2022) procedure was implemented.

In terms of overall analyses of RTs and accuracy, the results of Experiments 1A and 1B showed a clear difference between tasks, with the dual-task procedure (i.e., Experiment 1B) being more difficult (i.e., showing longer RTs and lower accuracy) than the single-task procedure (i.e., Experiment 1A). There was, however, no significant semantic Stroop interference overall in either experiment. Delta-plot analyses, instead, revealed a more complex pattern: While in the single-task procedure, a (small) semantic Stroop interference effect emerged and was constant across quantiles, in the dual-task procedure the small semantic Stroop interference effect in the faster quantiles seemingly reverted to a 12 ms facilitation effect (with a 25 ms *SE*) in the slower ones.

The results of Experiments 2A and 2B showed a different pattern. Whereas the analyses of overall RTs replicated the task effect found in Experiments 1A and 1B, they also revealed a reliable semantic Stroop interference with no clear evidence of any modulation as a function of procedure (as indicated by the absence of a Task × Condition interaction in the joint analysis of Experiments 2A and 2B). Notably, delta-plot analyses revealed finer-grained differences in terms of the distributional profile of the semantic Stroop effect. In the-single task procedure (Experiment 2A), the semantic Stroop interference effect increased as a function of response latency, whereas in the dual-task procedure (Experiment 2B), a small effect emerged and remained constant across quantiles. In what follows, we begin by discussing the results of Experiments 2A and 2B, and then we move on to Experiments 1A and 1B and to the possible reasons underlying the differences between the two pairs of experiments. Before the discussion, a caveat is needed. Although we consider our Stroop manipulation involving color-associated words as a rather pure index of semantic interference, it might also trigger response conflict via the indirect activation of the set of response colors (e.g., Cohen et al., 1990; Roelofs, 2003; for a discussion, see Parris et al., 2022). To disentangle the exact contribution of semantic conflict to the effect we report, we would have needed a measure of response conflict. One possibility would be to use the same response button (e.g., z) for two color responses (e.g., red and blue) so that certain word-color combinations (e.g., *red* written in blue) would allow to isolate semantic conflict, and any difference between these and other incongruent combinations (e.g., *red* written in green, with red and green associated with different response buttons) would be an index of response conflict (for further details on this paradigm, see Parris et al., 2022). The above concern, however, may be in part mitigated by the fact that the semantic conflict triggered by color associated words has been shown to correlate with a purely semantic interference effect (the taboo interference), corroborating the notion of an authentic semantic origin of the interference generated by color-associated words (Scaltritti et al., 2022). Additionally, we deemed it important to maintain the same manipulation used in our previous investigations (Scaltritti et al., 2022; Sulpizio et al., 2022), as it generated the hypotheses at stake in the present research.

## Semantic interference effects with reduced proactive control (Experiments 2A and 2B)

Results of Experiment 2A show the classic semantic Stroop interference effect (Klein, 1964; see also Dalrymple-Alford, 1972) and replicate our previous studies in terms of the distributional profile of the interference effect (Scaltritti et al., 2022; Sulpizio et al., 2022), which is close to zero in the faster quantiles and becomes larger only in the slower ones (for a similar pattern, see also Hasshim et al., 2019). In line with previous studies adopting the *n*-back manipulation in combination with a Stroop paradigm (Kalanthroff et al., 2015; Soutschek et al., 2013), the dual-task procedure hindered the performance in the Stroop task, as indicated by the slower and less accurate responses in Experiment 2B versus 2A. This pattern may be ascribed to the load on the executive control system (e.g., Chatham et al., 2011; Owen et al., 2005), which would leave fewer resources to perform any concurrent task requiring selective attention (Lavie et al., 2004). Note, however, that in the overall analyses the load seems to have a comparable impact on both the condition posing more demands on selective attention (the semantically associated condition) and the control condition, as the semantic Stroop effect always appeared to be very small.

The critical finding from Experiments 2A and 2B concerns how the *n*-back task changed the distributional profile of the semantic Stroop interference effect. While in absence of any cognitive load the effect increased as a function of response latency, it remained constant across quantiles when a cognitive load was introduced. Although this pattern is partially different from our predictions, it appears to be informative on the nature of semantic Stroop interference. Taken together, in fact, the results of Experiments 2A and 2B suggest that semantic Stroop interference surfaces in two different ways depending on the availability of executive resources. When executive resources are virtually intact (as in the single-task procedure used in Experiment 2A), semantic Stroop interference surfaces as a sporadic phenomenon mostly bounded to slower responses (i.e., those that would reflect transient failures in proactive maintenance of the task goal; De Jong et al., 1999). Differently, when few executive resources are available (e.g., as in the dual-task procedure used in Experiment 2B), semantic Stroop interference emerges as a systematic phenomenon across the whole RT distribution (for generalized effects of working memory load across conditions during selective attention tasks, see also Jongen & Jonkman, 2011; Lavie et al., 2004; Spinelli et al., 2020). In this situation, the difficulty in proactively maintaining the task goal would not be restricted to the slower responses but would more generally occur across trials due to the general depletion of executive resources. Because of the reliance on reactive control, word reading would in fact systematically interfere with the color identification task and, as a result, semantic interference will be triggered across the whole RT distribution. This explanation builds on the idea that the trials in which processing is more efficient under typical conditions would be those affected the most by any detrimental effect caused by an interfering task (as the *n*-back task). In other words, trials in faster quantiles, which are less prone to interference in the typical (i.e., single-task) condition, are those suffering more for the presence of the second *n*-back task drawing executive resources away.

One observation seems however at odds with this explanation. In Experiment 2B, the semantic Stroop interference effect remained small across the whole RT distribution, with its largest magnitude being 5 ms in the second and third quantiles. In contrast, in the last quantile in Experiment 2A, the effect was as large as 17 ms (see Supplementary Table 2). If the working memory load manipulation used in Experiment 2B triggers systematic failures in proactive control (assumed to characterize the slowest responses of Experiment 2A) across trials, then the semantic Stroop interference effect detected across all the quantiles of Experiment 2B should have had a magnitude close to the one found in the slowest responses in Experiment 2A (i.e., around 17 ms). As this was not the case, a somewhat more complex explanation seems to be required.

One possibility is that, in the dual-task procedure, participants tended to postpone responses to the Stroop task, for example, to gain some extra time for processes related to the *n*-back task. During this postponement, while finishing up encoding, updating, and/or refreshing processes relevant to the n-back task, the participant would also start working out the Stroop stimulus. Thus, even though in the dual-task procedure participants would have fewer executive resources to proactively deal with interference phenomena and would thus face enhanced costs, they would also be able to absorb part of those costs by the time they are ready to respond to the Stroop task. Overall, this idea would seem to do a decent job explaining why, while semantic Stroop interference emerged across the whole distribution of RTs in Experiment 2B (because of a systematic failure to proactively maintain the task goal), the magnitude of the effect remained small (because postponement of responses to the Stroop task allowed participants to offset the impact of semantic interference).

This interpretation warrants some caution, as it assumes that part of the resolution of the semantic Stroop task can occur in parallel while the system is engaged in another task requiring a large part of the available executive resources. However, experimental evidence suggests that Stroop effects are not absorbed within slowdowns induced by dual-task procedures (e.g., Fagot & Pashler, 1992), consistent with the notion that the resolution of Stroop phenomena hinges on limited central resources. Albeit the semantic Stroop has not been specifically tested in these types of experiments, evidence has been produced for similar semantically triggered Stroop phenomena, such as the emotional Stroop effect (Janczyk et al., 2014), casting concerns on the notion that part of the semantic Stroop effect triggered in the present experiment may have been resolved in parallel with the *n*-back task. A second possible issue contributing to the reported pattern might be related to interindividual variability in working-memory capacity, which has been shown to modulate proactive control engagement (e.g., Lin et al., 2022; Richmond et al., 2015). Future studies might further validate our findings by also taking into account participants’ working memory capacity.

In any case, our data seem to suggest that, compared with the standard task configuration (Scaltritti et al., 2022; Sulpizio et al., 2022), a condition of *reduced* proactive control may alter the distributional profile of the semantic Stroop effect, by making it constant across quantiles. To support this interpretation, we sought converging empirical support by exploring experiments building on the manipulation of the proportion of trials involving conflict. This type of manipulation has been linked to modulations of proactive/reactive control: Lists of trials with a low proportion of conflicting stimuli are assumed to bias participants towards reactive control (for a review, see Bugg & Crump, 2012).^3^

We examined the distributional pattern of the semantic Stroop effect when the trial list involves low proportion of conflicting stimuli. As this context is expected to reduce proactive control, the semantic Stroop effect should display a distributional pattern akin to the one reported in Experiment 2B, in which the high-working-memory load depleted the resources available to support proactive control. Notably, Kinoshita et al. (2018) conducted two experiments featuring a manual Stroop task with experimental lists mostly (75%) composed of (nonletter) neutral trials (e.g., ### in red). In their Experiment 2, the semantic Stroop effect was measured exactly as we did in our experiments, that is by comparing color-associated words (e.g., *lemon* written in blue) with color-unrelated words (e.g., *mercy* written in blue). In their Experiment 4, instead, the semantic Stroop effect was measured by comparing color names not part of the response set (e.g., *green* written in red) with control words (e.g., *twice* written in red). In terms of mean RTs, Experiment 2 produced no evidence for the semantic Stroop effect being modulated by list type, whereas in Experiment 4 the semantic Stroop effect was smaller in the low- than in high-neutral proportion list. We reanalyzed the data of these two experiments to investigate the underlying delta plots. The results (for details, see Supplementary Materials 2) paralleled those we obtained in Experiment 2B, with the semantic Stroop interference surfacing, with a similar size, across the whole RT distribution. Although these results need to be taken with some caution, they offer a further line of evidence that, in a manual semantic Stroop task, little use of proactive control is associated with a specific type of distributional profile, in which semantic interference occurs more homogeneously across the whole RTs distribution.

## The influence of the timing of the events on the semantic Stroop interference (Experiment 1A and 1B)

Moving to the first set of experiments, Experiment 1A produced a small semantic Stroop interference effect which reached significance only in the delta-plot analysis with no evidence that the effect changed as a function of response latency. In Experiment 1B, the semantic Stroop interference effect reversed in the slowest quantiles, with faster responses to the semantically associated condition than to the control one. Note that the reversal of the effect appears to be particularly difficult to explain with reference to selective attention mechanisms, because even an optimal filtering of the distractor would produce, at best, an elimination, and not a reversal, of interference effects (for discussions, see, e.g., Weissman et al., 2015). Thus, even though we did obtain a reversed semantic Stroop interference effect (i.e., a facilitation effect) in the slowest quantiles, we presume that this reversal is due to noise, with the elimination of the effect being the most likely pattern, possibly indicating that the offsetting of interference effects hypothesized above for the dual-task procedures might be so strong in the slowest quantiles to eliminate interference effects. Although tentative, this interpretation seems to be the most cautious and reasonable one, as the alleged effect in the fifth quantile is characterized by a very large error bar ranging from (approximately) +10 ms to (approximately) −40 ms, thus encompassing 0 and suggesting that the 12 ms difference may just be a nominal one.

With respect to Experiment 1A (the single-task procedure), one may wonder why the semantic Stroop effect was elusive and did not increase across quantiles, as reported in previous investigations (Scaltritti et al., 2022; Sulpizio et al., 2022) featuring only minor differences with respect to the current implementation. We can only present tentative and post hoc speculations, as the findings from Experiment 1A were admittedly unexpected. A posteriori, we reasoned that Experiment 1A involved a longer ISI as well as a longer interval for response and the latter feature might have pushed participants to use a more relaxed response criterion, which in turn would have left them more time to resolve the interference before delivering the response. Albeit previous studies reported increased Stroop interference for longer response-stimulus intervals (RSI), Augustinova et al. (2018) showed that this is not the case for semantic conflict indexed via the manipulation of color-associated words, suggesting that the different RSI across Experiment 1A and 1B may not be the critical parameter underlying the discrepancies in the results. Differently, the time allotted for the responses may influence response criteria and speed-accuracy tradeoffs, with longer response intervals biasing participants towards more conservative responses. To empirically assess our speculation, we ran post hoc analyses with mixed-effects models contrasting RTs and error rates between Experiment 1A (the single-task procedure with the slower trial sequence) and Experiment 2A (the single-task procedure with the faster trial sequence; experiment was the only fixed effect in this model, with random intercepts for participants and target colors). The analysis revealed a main effect of experiment, χ^2^(1) = 10.87, *p* < .001, *b* = −44.86, *SE* = 13.44, *t* = −3.33, with participants being faster in Experiment 2A than in Experiment 1A. Notably, the same analysis on response accuracy showed that participants were *less* accurate in Experiment 2A than in Experiment 1A, χ^2^(1) = 16.48, *p* < .001, *b* = −0.48, *SE* = 0.11, *z* = −4.16. Such a speed–accuracy trade-off supports the view that, when more time is available, participants may adopt a more conservative approach to optimize their performance to the current task conditions (e.g., Jones et al., 2002; Verbruggen & Logan, 2009; for a similar logic, but with manipulations intended to create a speed accuracy trade-off, see, e.g., Rinkenauer et al., 2004). As a result of this more conservative approach, a semantic interference effect would be less likely to emerge, even in the slowest responses. The take-home message is that the pattern of increasing semantic Stroop interference that we reported in our previous works (Scaltritti et al., 2022; Sulpizio et al., 2022) and in the current Experiment 2A may be susceptible to subtle changes in the trial procedure and may be best observed when the procedure creates sufficient time pressure. It is further important to note that, in their manual Stroop task under low working memory load, Kalanthroff et al. (2015) reported a sizable Stroop effect using the same stimulus presentation time and ISI duration we used in Experiment 1A. The asymmetry between Kalanthroff et al.’s and our results may be due to three different reasons: The size of the semantic conflict, the strength of the semantic activation in the classic versus semantic Stroop paradigm, and the presence of a potential contingency-learning confound in Kalanthroff et al.’s (2015) study. With respect to the size of the semantic conflict in the manual Stroop paradigm, it is well established that this effect (around 10–12 ms; e.g., Kinoshita et al., 2018, Experiment 2; Scaltritti et al., 2022; Sulpizio et al., 2022) is considerably smaller than the one elicited by the classic Stroop contrast (e.g., *red* written in blue vs. *blue* written in blue) investigated by Kalanthroff et al. (approximately 50 ms in that particular experiment, and often larger). The main reason for this large difference is that, while the semantic Stroop interference mostly reflects semantic conflict (e.g., Augustinova et al., 2018; Neely & Kahan, 2001), the classic Stroop interference is the result of multiple concurrent types of conflicts (i.e., semantic, task, and, in some cases, response conflict, e.g., Parris et al., 2022).

Regarding the strength of semantic activation, in the semantic Stroop effect the color is just one of the concept’s semantic features activated by the written word (e.g., yellow when *lemon* is presented), whereas in the classic Stroop manipulation the color is itself the full concept activated by the written word (e.g., yellow when *yellow* is presented). Therefore, assuming a spreading of activation mechanism, the concept of yellow will receive weaker activation in the case of *lemon* than in the case of *yellow*.

Finally, the presence of a contingency-learning confound (Schmidt & Besner, 2008) in the experimental design of Kalanthroff et al. (2015) may have contributed to the pattern of results reported therein. Specifically, each of the color name distractors used (i.e., the Hebrew translation equivalents for *red*, *blue*, *green*, and *yellow*) appeared in stimuli that required the color response congruent with the distractor (e.g., for the word *red*, the “red” response) 50% of the time and each of the three incongruent color responses (e.g., for the word *red*, the “blue” response) 16.67% of the time. In contrast, the neutral letter string distractor appeared in stimuli that required each of the four color responses 25% of the time. As a result, participants could learn to associate each color name with its congruent response (e.g., the word *red* and the “red” response) whereas they could not associate the neutral letter string with any color response. This confound might have facilitated congruent stimuli with respect to incongruent ones, but, most importantly, also with respect to neutral ones.

It is worth underlining that Kalanthroff et al. obtained an interaction between cognitive load and the Stroop *facilitation* effect, that is, the contrast between congruent and neutral stimuli. The importance of this contrast involving a contingency learning confound is clear when one considers the finding that load manipulations reduce contingency learning effects (Schmidt et al., 2010; Spinelli et al., 2020). Based on that finding, it can be assumed that in the low-load condition of Kalanthroff et al.’s experiment, contingency learning was not impaired and boosted congruent stimuli, pushing the congruent-neutral contrast towards facilitation (i.e., congruent faster than neutral). With high load, on the other hand, contingency learning was impaired and could not boost congruent stimuli, thus providing little or no bias towards facilitation in the congruent-neutral contrast. As a result, an interaction would arise. Note that this should not be the case for our experiments, as these involved no contingency learning confound. In sum, for all these reasons, Kalanthroff et al.’s load manipulation might have interacted with the Stroop effect, whereas ours did not.

## Load-induced modulations of semantic Stroop effects in models of conflict resolution

The modulation induced by working memory load on the distributional profile of the semantic Stroop effect that we report may be informative for Kalanthroff et al.’s (2015, 2018) model of conflict resolution. Akin to previous proposals (De Pisapia & Braver, 2006), the model assumes a task-demand layer that includes units for each of the possible tasks available for a given stimulus (e.g., color naming and word reading). These task units are linked to (color and word input units within) the input layer by means of excitatory bidirectional connections. Conflict between task units would inhibit the response layer via a unidirectional connection. Finally, a proactive control unit, whose activation reflects the amount of proactive control (and inversely, reactive control) being engaged, would modulate the ability of the task-demand layer, with which the proactive control unit is unilaterally connected, to deal with task conflict. When proactive control is high (and reactive control is low), task conflict can be more easily resolved within the task-demand layer, without any inhibition being forwarded to the response layer. In contrast, when proactive control is low (and reactive control is high), there would be less advance preparation for the conflict arising in the task-demand layer and the response layer will be inhibited until task conflict is resolved, slowing down latencies. Kalanthroff et al. (2015, 2018) assume that, among other things, proactive control may be low because of a high working memory load. In these circumstances, task conflict would be harder to deal with and latencies will slow down as a result of the response layer being inhibited.

What is important to note, however, is that Kalanthroff et al.’s model predicts that a working memory load manipulation should similarly affect any condition associated with readable stimuli: Under low-proactive control conditions, any readable word (being either congruent (e.g., *red* in red), neutral (e.g., *table* in red), or incongruent (e.g., *green* in red)) should produce task conflict. The consequence would be an additive pattern between a working memory load and a Stroop manipulation involving only readable stimuli. Although the working memory load would slow down processing overall, the consequent inability to use proactive control in that situation should similarly affect congruent, readable neutral, and incongruent stimuli, as all of them would produce a similar degree of task conflict via bottom-up activation (e.g., Goldfarb & Henik, 2007; Kalanthroff et al., 2015).

Although the overall semantic Stroop effect of our Experiments 2A and 2B is in line with the model’s prediction (and with the findings reported by Kalanthroff et al., 2015), the patterns we report in the delta-plot analyses suggest a slightly more complex picture. As the reduction of proactive control has the same impact on any readable stimulus (including our semantically associated and neutral-readable conditions), the model predicts the same distributional profiles in both the single- and the dual-task procedure, with a mere shift towards slower ranges of latencies in the latter, due to a (constant) time needed for the resolution of the increased task conflict. Although it is important to acknowledge that Kalanthroff’ et al.’s (2015) model does not make explicit predictions on RT distributions, which are possibly beyond the scope of the model itself, we would note that the modulation of the distributional profiles induced by the working memory load in the present experiments suggests that, depending on the response latency, working memory load may have a different impact on neutral-readable and semantically-associated stimuli. Indeed, as already noted above, the impact of working memory load on the contrast between neutral-readable and semantically associated stimuli seemed to be more pronounced in the faster responses, which typically produce no semantic Stroop interference in baseline conditions (i.e., in the single-task procedure) whereas they produce a reliable (albeit small) semantic Stroop interference in conditions of high working memory load (i.e., in the dual-task procedure).

## Conclusions

In conclusion, our results clearly show that, provided that the experiment exerts sufficient time pressure, semantic Stroop interference is a reliable, albeit small, phenomenon that tends to grow in slower quantiles. Importantly, such interference seems to depend, to some extent, on executive resources: The lack of such resources produces a change in the distributional profile of the semantic Stroop interference, which we ascribe to the difficulty in the use of effortful proactive control in maintaining the task goal. This difficulty would be rather general, with a widespread influence across conditions (control and semantically associated words) and RT distributions, although the processing cost associated with this difficulty might be partially absorbed by the time a response is emitted. Finally, the emergence of semantic Stroop interference seems to be highly sensitive to subtle variations in task conditions. Taken together, our results shed light on the interaction between semantic processing and executive resources, making the case for the importance of studying the interaction between the core processes of visual word recognition and executive control more generally.

### Supplementary Information

Below is the link to the electronic supplementary material.

Supplementary file1 (DOCX 113 KB)

## Data Availability

All data are available on the Open Science Framework (https://osf.io/bzmr2/).

## Footnotes

1 Note that effect sizes were not calculated for accuracy analyses, since, as stated by the authors of the library, “for generalized linear models (Poisson, Logistic…), where the outcome is never on an arbitrary scale, estimates themselves *are* indices of effect size!” (https://cran.r-project.org/web/packages/effectsize/vignettes/from_test_statistics.html#in-linear-mixed-models).

2 In this and all the following analyses, we also tested the inclusion of a quadratic orthogonal polynomial term. Although such term always increased the models’ fit, it never interacted with the critical condition of interest (i.e., the semantic Stroop effect). Therefore, we opted for reporting the analysis with the linear term only.

3 Note that this type of manipulation is likely weaker than a working memory manipulation, as participants assumed to have the resources necessary to use proactive control in all situations (e.g., high-working-memory capacity participants) tend to use proactive control independently of the list context (e.g., Hutchison, 2011; Kane & Engle, 2003).

## Code availability

Scripts for the analyses are available from the first author upon request.
