# Supplementary material for: Semantic Stroop interference is modulated by the availability of executive resources: Insights from delta-plot analyses and cognitive load manipulation
Source: Mem Cognit. 2024 Mar 26;52(6):1422–38. doi: 10.3758/s13421-024-01552-5 (PMC11362381; doi:10.3758/s13421-024-01552-5)
Supplement: Supplementary file 1 — Supplementary file1 (DOCX 113 KB) [file 13421_2024_1552_MOESM1_ESM.docx]

**Supplementary Materials 1**

**Supplementary Table 1**

*Mean RTs for condition for each quantile in Experiments 1A (single-task procedure) and 1B (dual-task procedure).*

| Condition |  | Quantiles | | | | |
| --- | --- | --- | --- | --- | --- | --- |
|  |  | 1 | 2 | 3 | 4 | 5 |
|  |  | Single Task | | | | |
| Semantic |  | 474 | 574 | 654 | 765 | 1056 |
| Control |  | 473 | 569 | 649 | 757 | 1049 |
| Difference |  | 1 | 5 | 5 | 8 | 7 |
|  |  | Dual Task | | | | |
| Semantic |  | 577 | 694 | 801 | 947 | 1300 |
| Control |  | 574 | 691 | 800 | 954 | 1312 |
| Difference |  | 3 | 3 | 1 | -7 | -12 |

**Supplementary Table 2**

*Mean RTs for condition for each quantile in Experiments 2A (single-task procedure) and 2B (dual-task procedure).*

| Condition |  | Quantiles | | | | |
| --- | --- | --- | --- | --- | --- | --- |
|  |  | 1 | 2 | 3 | 4 | 5 |
|  |  | Single Task | | | | |
| Semantic |  | 461 | 556 | 628 | 723 | 944 |
| Control |  | 460 | 552 | 620 | 707 | 927 |
| Difference |  | 1 | 4 | 8 | 16 | 17 |
|  |  | Dual Task | | | | |
| Semantic |  | 556 | 658 | 743 | 854 | 1081 |
| Control |  | 552 | 653 | 738 | 852 | 1082 |
| Difference |  | 4 | 5 | 5 | 2 | -1 |

**Supplementary Materials 2**

**Delta-plot analyses of Kinoshita et al.’s (2018) data**

Before reporting our delta-plot analyses, we briefly summarize Kinoshita et al.’s experiments. For all the details the reader may directly refer to the original article.

**Experiment 2**

Three experimental conditions were used: color associated words – i.e., words associated with one of the response colors (e.g., *lemon*) –, control words – i.e., words not associated with any color (e.g., *mercy*) –, and neutral strings – i.e., strings of #. Stimuli were presented in four colors: Red, yellow, green, and blue, and participants were asked to categorize the color in which the stimuli were presented manually, i.e., by button press.

Two different lists were used. In the high-neutral proportion list, neutral stimuli occurred on 75% of the trials, whereas in the low-neutral proportion list, neutral trials appeared on 25% of the trials. Note that the two lists featured different proportions of the different types of conflict involved. Specifically, in the 25%-neutral condition, 25% of the trials (the neutral ones) involve strings of non-alphabetic characters (######), which do not trigger any type of conflict. Next, 37.5% of the trials involve color-unrelated words (e.g., MERCY), which trigger only task conflict. Finally, the remaining 37.5% of the trials involve color-associated words (e.g., SKY), which trigger both task and semantic conflict. In contrast, in the 75%-neutral condition, 75% of the trials are neutral (i.e., #####) and do not trigger any type of conflict. Next, 12.5% of the trials involve color-unrelated words (e.g., MERCY) and thus trigger only task conflict. Finally, the remaining 12.5% of the trials are color-associated words (e.g., SKY) and trigger both task and semantic conflict.

In sum, the 25% and the 75% neutral condition clearly involve different proportions of trials in which any type of conflict occurs (i.e., 75% in the 25%-neutral condition vs. only 25% in the 75% neutral condition) and in which semantic conflict occurs (i.e., 37.5% of trials in the 25%-neutral condition vs only 12.5% of trials in the 75%-neutral condition). On the basis of these characteristics, the critical list for our hypothesis (i.e., that a reduction of proactive control is associated with a semantic interference effect occurring throughout the RTs distribution) was the high-neutral proportion one. The reason is that, since proactive control is assumed to be positively correlated with the frequency of conflict (either semantic, task, or both; see, e.g., Botvinick et al., 2001; Kalanthroff et al., 2018), a list in which conflict of either type is infrequent, such as Kinoshita et al.’s high-neutral proportion list, would provide little reason for participants to maintain effortful proactive control and should bias them towards reactive control. Therefore, our delta-plot analyses focused on the high-neutral proportion list. Differently, the relative occurrence of conflicting trials in the low-neutral proportion list falls in a somewhat grey area between the standard task configuration (50% color-associated words, 50% color-unrelated control words) used in our experiments and a low-conflict list such as Kinoshita et al. high-neutral proportion list. As this complicates any clear-cut comparison and interpretation, we refrain from considering the low-neutral proportion condition further.

We ran delta-plot analyses identical to those conducted in our experiments. Within each participant and within each condition, RTs were partitioned into 5 quantiles: The first quantile included the fastest 20% of responses, the second quantile the next fastest 20%, and so on, until the fifth quantile, which included the slowest 20% of the responses. Changes in semantic Stroop effects as a function of response latency were assessed by entering the variables Stroop condition (color-associated vs neutral), quantile (considered as numerical), and their interaction as fixed effects in the statistical model. Participants and target colors were included as random intercepts.

Supplementary Table 3 reports mean RTs for each condition in each quantile. The interference effect across quantiles is showed in Supplementary Figure 1a. The analysis showed simple effects of Stroop condition (*χ^2^* (1) = 8.47, *p* = .003, *b* = -16.95, *SE* = 5.82, *t* = -2.91) and quantile (*χ^2^* (1) = 2004.19, *p* < .001, *b* = 126.41, *SE* = 2.03, *t* = 61.99), but no interaction (*χ^2^* (1) = 1.84, *p* = .17) even though the last quantile showed the largest semantic interference effect (46 ms, but do note the large error bar associated with it in Supplementary Figure 1a).

**Supplementary Table 3**

*Mean RTs for condition and quantile in Kinoshita et al.’s (2018) Experiment 2, high-neutral proportion list.*

| Condition |  | Quantiles | | | | |
| --- | --- | --- | --- | --- | --- | --- |
|  |  | 1 | 2 | 3 | 4 | 5 |
| Semantic |  | 462 | 567 | 648 | 743 | 1023 |
| Control |  | 450 | 555 | 640 | 740 | 977 |
| Difference |  | 7 | 12 | 8 | 3 | 46 |

**Experiment 4**

Three experimental conditions were used: One with color names (i.e., *green*, *yellow*, *grey*, and *pink*) that were not the response colors, one with control noncolor words (*twice*, *winner*, *grip*, and *thank*), and one with neutral strings – i.e., strings of #. Stimuli were presented in four colors: Red, blue, orange, and white. The other experimental details paralleled those of Experiment 2.

The same statistical approach adopted for Experiment 2 was also adopted here, and we again focused on the high-neutral proportion list. Supplementary Table 4 reports mean RTs for each condition in each quantile. The interference effect across quantiles is showed in Supplementary Figure 1b. The results show simple effects of Stroop condition (*χ^2^* (1) = 46.24, *p* < .001, b = -44.67, *SE* = 6.51, t = -6.85) and quantile (*χ^2^* (1) = 1382.73, *p* < .001, b = 105.27, *SE* = 2.12, t = 49.45), but no interaction (*χ^2^* (1) = 0.09, *p* = .76) (and note that this time, the last quantile showed the smallest semantic interference effect – 22 ms – contrary to what was found for Kinoshita et al.’s (2018) Experiment 2).

**Supplementary Table 4**

*Mean RTs for conditions for each quantile in Kinoshita et al.’s (2018) Experiment 4 – high-neutral proportion list*.

| Condition |  | Quantiles | | | | |
| --- | --- | --- | --- | --- | --- | --- |
|  |  | 1 | 2 | 3 | 4 | 5 |
| Semantic |  | 481 | 573 | 636 | 726 | 922 |
| Control |  | 441 | 528 | 589 | 669 | 900 |
| Difference |  | 40 | 45 | 47 | 57 | 22 |

**Supplementary Figure 1**

*Results of the delta-plot analyses for Kinoshita et al.’s (2018) Experiments 2 and 4, high-neutral proportion lists.*


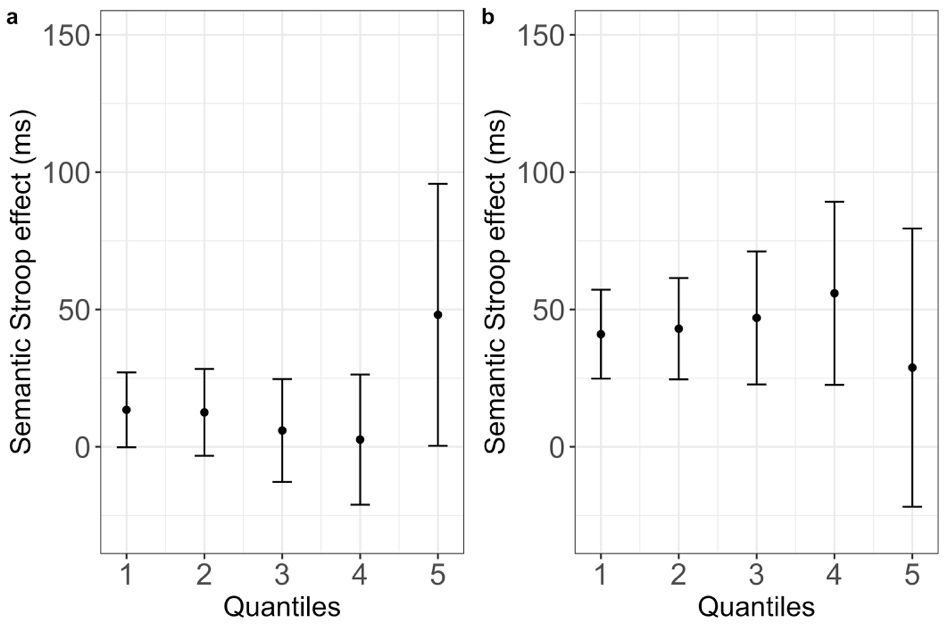


*Note.* Mean semantic Stroop effect (Experiment 2: color-associated – control; Experiment 4: non-response-set color names – control; y axis) as a function of trial quantile (x axis) for the high-neutral list in Experiment 2 (panel a) and Experiment 4 (panel b). Points represent empirical means, and error bars reflect corresponding 95% confidence intervals.

**References**

Botvinick, M. M., Braver, T. S., Barch, D. M., Carter, C. S., & Cohen, J. D. (2001). Conflict monitoring and cognitive control. *Psychological Review*, *108*, 624-652.

Kalanthroff, E., Davelaar, E. J., Henik, A., Goldfarb, L., & Usher, M. (2018). Task conflict and proactive control: A computational theory of the Stroop task. *Psychological Review, 125*, 59-82.
